# Wearables measuring electrodermal activity to assess perceived stress in care: a scoping review

**DOI:** 10.1017/neu.2023.19

**Published:** 2023-03-24

**Authors:** Agata Klimek, Ittay Mannheim, Gerard Schouten, Eveline J. M. Wouters, Manon W. H. Peeters

**Affiliations:** 1 School for Allied Health Professions, Fontys University of Applied Sciences, Eindhoven, The Netherlands; 2 School for Information & Communication Technology, Fontys University of Applied Sciences, Eindhoven, The Netherlands; 3 Tranzo, School of Social and Behavioural Sciences, Tilburg University, Tilburg, The Netherlands

**Keywords:** psychological stress, wearable electronic devices, galvanic skin response, problem behaviour, mental disorders

## Abstract

**Background:**

Chronic stress responses can lead to physical and behavioural health problems, often experienced and observed in the care of people with intellectual disabilities or people with dementia. Electrodermal activity (EDA) is a bio-signal for stress, which can be measured by wearables and thereby support stress management. However, the how, when and to what extent patients and healthcare providers can benefit is unclear. This study aims to create an overview of available wearables enabling the detection of perceived stress by using EDA.

**Methods:**

Following the PRISMA-SCR protocol for scoping reviews, four databases were included in the search of peer-reviewed studies published between 2012 and 2022, reporting detection of EDA in relation to self-reported stress or stress-related behaviours. Type of wearable, bodily location, research population, context, stressor type and the reported relationship between EDA and perceived stress were extracted.

**Results:**

Of the 74 included studies, the majority included healthy subjects in laboratory situations. Field studies and studies using machine learning (ML) to predict stress have increased in the last years. EDA is most often measured on the wrist, with offline data processing. Studies predicting perceived stress or stress-related behaviour using EDA features, reported accuracies between 42% and 100% with an average of 82.6%. Of these studies, the majority used ML.

**Conclusion:**

Wearable EDA sensors are promising in detecting perceived stress. Field studies with relevant populations in a health or care context are lacking. Future studies should focus on the application of EDA-measuring wearables in real-life situations to support stress management.

## Summation

- The number of studies regarding wearable sensors measuring EDA and perceived stress are increasing, with more field studies being conducted. It is shown that this technology can be used to assess perceived stress.
- Studies that show a relation between EDA measured by wearables and perceived stress sometimes use sophisticated techniques such as machine learning. However, the fact that data processing and analyses are mainly being done offline hampers the practical usability in the field.
- Conclusions regarding their application in a healthcare context are limited, as this context is understudied.

## Considerations

- The methodology to process the EDA signal and relate it to perceived stress varies greatly across studies, hampering comparability between studies.
- Multiple studies include other bio-signals besides EDA features in relation to stress with the same statistical model. It was not always possible to draw conclusions about the independent relationship between EDA (without other bio-signals) and stress.
- No solid conclusion can be drawn regarding usability and adoption in healthcare populations, as studies in naturalistic healthcare contexts are scarce.

## Introduction

Stress plays a significant role in our society and is described by the World Health Organization as the ‘Health Epidemic of the 21^st^ Century’ (Fink, 2017). Selye described stress as ‘the disturbance of the body’s homeostasis triggered by an in- or external factor’ (Selye, 1956). Stress is seen as a broad concept, including both a physical and mental load (Van Houdenhove, 2007; Dhabhar, 2018). A stressful event involves a stimulus (stressor), which triggers a response in the brain (stress perception), which activates physiological fight or flight systems in the body (stress response). The stressor can be external or internal, physical (e.g. physical exertion or a painful stimulus) or psychological (e.g. work pressure). When stress is too intense or prolonged for a person, it can also have negative consequences and lead to psychological and/or physical health conditions, such as (but not limited to) cardiovascular problems (Steptoe & Kivimäki, 2012), anxiety disorders (Van Houdenhove, 2007) and persistent physical symptoms (Deary *et al*., 2007). This leads to major healthcare concerns in all types of populations in which stress management plays an important role, such as people with stress-related physical problems (Deary *et al*., 2007). Additionally, stress management is of utmost important in people living with dementia or intellectual disability (Melander *et al*., 2017), as stress-related behavioural problems are frequently experienced.

Up to 90% of the people with dementia present challenging behaviours at some point (Devshi *et al*., 2015), whereby ‘apathy, depression, irritability, agitation and anxiety, […] are the most frequent symptoms’(Cerejeira *et al*., 2012). Stress is considered to be an antecedent leading to challenging behaviour, such as agitation (Ballard *et al*., 2001; Melander *et al*., 2018). Agitation is defined as the attempt of expressing unmet needs and unexpressed emotions as a response to in- or external stimuli (Kong, 2005) and can negatively affect the health of both patients and caregivers (Ornstein & Gaugler, 2012). Besides dementia, other psychopathological and/or intellectual disabilities are often associated with difficulty to understand stress-induced behaviour. Children with autism spectrum disorders (ASDs) have shown to have a prevalence of 56 to 94% to present challenging behaviours (Matson *et al*., 2007). Such challenging behaviours seem not to decrease with time, which is why ‘an optimized intervention’ could help to increase their quality of life (Rattaz *et al*., 2017). Early detection of stress onset using wearable sensors may be an effective way to (self-)manage stress in order to treat or prevent health issues (Zangróniz *et al*., 2017). Additionally, it facilitates the management of stress before it effects behaviour and may assist caregivers to mitigate and cope with stress-related challenging behaviour (Melander *et al*., 2017). However, it is still not commonly known how accurate wearables are in measuring and predicting stress, and how they can be utilised in caregiving.

Different bio-signals, which can be measured by wearables, are essential for the detection of acute and/or chronic stress. Physiological parameters that are influenced by the sympathetic nervous system (SNS) are as follows: 1) heart activity, 2) electrodermal activity (EDA), 3) muscle activity, and 4) pupil diameter (Wijsman *et al*., 2011). Sierra *et al*. (2011) claim that EDA and heart rate (HR) are the two most important parameters enabling a high potential detection of acute stress (Sierra *et al*., 2011). EDA, also known as galvanic skin response (GSR) or Skin Conductance, is the activity resulting from constant changes of the electrical properties of the skin. Sweating leads to a continuous change of the electrical properties of the skin. These electrical changes can be measured by placing two electrodes on the skin and give an insight into a person’s emotional state, psychological or physiological arousal, which indicate stress (Kurniawan *et al*., 2013).

Changes in EDA are considered to be one of the best indicators for real-time stress (Healey & Picard, 2005). However, the measurement of EDA still includes technological and acceptance challenges. Electrodes are commonly placed on the fingers and wrist, although it is still debated which technology and bodily location is appropriate, with respect to (technical) validity (van Dooren *et al*., 2012; Payne *et al*., 2013). Importantly, validity can be assessed on different levels (i.e. on the ‘raw’, ‘parameter’ or ‘event’ level, see (van Lier *et al*., 2020). However, bodily location may also influence the acceptance of the wearer (Peeters *et al*., 2021).

Different methods, protocols and systems which enable real-time stress detection by measuring biological signals are currently studied (Minguillon *et al*., 2018). Wearable sensors are of great value due to their ability to measure non-invasively, easily and wireless (Hanson *et al*., 2009) and have the potential to support psychosocial approaches to manage challenging behaviours and thereby reduce pharmacological treatment (Foebel *et al*., 2015; Berg-Weger & Stewart, 2017). The application of wearables is steadily increasing, and technology is constantly evolving. The integration of devices such as wearables in the healthcare department is crucial. Furthermore, these tools are offering great opportunities for patients and their caregivers in terms of monitoring vital signs on a daily life basis and therapy provision (Rienzo *et al*., 2005). However, today’s wearable devices still require improvements regarding data collection, as well as increasing usability in ambulatory settings (Posada-Quintero & Chon, 2020). As the majority of the studies are studying wearables in the lab, there is a lack of use in real-life situations.

A review summarising scientifically studied wearable technologies measuring EDA to detect and predict stress is currently missing. In order to develop a reliable transparent real-time stress detection system, it is important to collect fundamental knowledge about the current application and accuracy of using EDA measurements in daily life related to perceived stress. The goal of the current scoping review is therefore to create an overview of existing wearable devices used for measuring and monitoring EDA for stress detection. Such an overview will help researchers and developers to optimise stress detection using EDA, aiming for early stress recognition to support healthcare. To this end, the following three research questions are formulated:

1. Which wearable devices are scientifically studied in relation to perceived stress?
2. How are those wearables studied, regarding the research populations, measured bio-signals, which stressors (induced or observed) where studied, and in what kind of circumstances (lab vs. field studies)?
3. What is reported about the relationship between the EDA measurement of the wearable with perceived stress or stress-related (e.g. challenging) behaviours?

## Methods

As the goal of this study is broad and aims to identify what is the most up-to-date knowledge regarding wearables measuring EDA, a scoping review was deemed as the most suitable method (Arksey & O’Malley, 2005; Levac *et al*., 2010; Munn *et al*., 2018; Tricco *et al*., 2018). To assure that the most relevant studies were included, systematically reviewed and summarised, we followed the checklist of the Preferred Reporting Items for Systematic reviews and Meta-Analyses extension for Scoping Reviews (PRISMA-ScR) (Tricco *et al*., 2018).

### Search strategy

As the topic of this study strongly relates to healthcare as well as technological innovation, the databases used for this scoping review were PubMed, Medline, ACM digital and Web of Science to assure the relevance of the search.

The search string was composed of the main keywords ‘Electrodermal Activity’, ‘Technological tools’, ‘Measurement’ and ’Stress’. The search strategy was not case-sensitive, and keywords were paired together with the Boolean connector ‘AND’ and their synonyms with ‘OR’ (Table 1). An asterisk (‘*’) was used for words starting with the same letters but having a different ending. Separate words, belonging together, have been searched for by inserting quotation marks (‘…’) (Table 1).

**Table 1.** Set of keywords and related synonyms

Keyword	Synonym
Electrodermal activity	Galvanic skin response, skin conductance, EDA, GSR
Technological tools	Wearable*, sensor*, wireless, electronic devices, psychophysiological devices, psycho-physiological devices
Measurement	Detect*, measure*, measuring, monitor*
Stress	Stress, stress*, behavioral/behavioural psychological symptoms in dementia, BPSD, distress, challenging behavior/behaviour, arousal, agitation, aggression, disinhibition, apathy, dementia, Alzheimer, psychiatry, autism, intellectual disability, anxiety, aberrant motor behavior/behaviour

### Inclusion and exclusion criteria

This scoping review included peer-reviewed full-text articles written in English and studies that have been conducted on humans. To be included, articles were required to describe the measurement of EDA measured by a wearable (i.e. wireless) sensor, related to perceived stress. In this study, we considered perceived stress as an affective (i.e. emotional) state with a negative valence (Posner *et al*., 2005; Du *et al*., 2018). Studies were included when perceived stress was measured using a subject’s self-report or by observations of stress-related behaviour. Only original research (including original data collection) was included. Opinion papers were excluded from this research, because their level of scientific evidence was considered inadequate for the purpose of this study. Literature reviews were not included because the study performed was not a meta-analysis of other literature reviews. Finally, as the evolution of wearable technology has increased remarkably since 2013 (CCS Insight, 2016; Statista, 2017), all articles published before 2012 were excluded (Table 2).

**Table 2.** In- and exclusion criteria

Inclusion criteria	Exclusion criteria
Peer-reviewed English articles Studies based on humans Including the assessment of perceived stress or stress-related (e.g. challenging) behaviours Original research articles Describing usage of wearables, measuring electrodermal activity Articles published before December 2022	Articles published before 2012

### Study selection

The final search was conducted on November 26 2022. Duplicates were removed using EndNote X9, and Covidence, a systematic review software (https://www.covidence.org/), was used to perform the study selection. Articles were first screened by title and abstract and by full text in the second phase by at least two independent researchers. The first author (AK) screened all papers, and the second assessment was divided between all other co-authors (IM, GS, EW, and MP). In case of a conflict, a third reviewer was involved. Calibration sessions were held with all authors in order to iteratively refine the selection process and solve ambiguities.

### Data extraction

Relevant data were extracted by at least two independent researchers and compared. AK extracted all the articles, and the other co-authors (IM, GS, EW, and MP) extracted one-fourth of the total number of the included studies. Whenever a conflict was present, the reviewers met to discuss until agreement was reached. The data extraction table included author(s), publication year, study’s purpose, the wearable used, measured bio-signals, whether the study was done in the lab or in practice, type of stressor (induced or observed), population included, number of subjects, EDA features processed, whether the data were processed real time or post-processed, perceived stress assessment, and finally the main results regarding the relationship between EDA and perceived stress or stress-related behaviours.

## Results

**Fig. 1.**
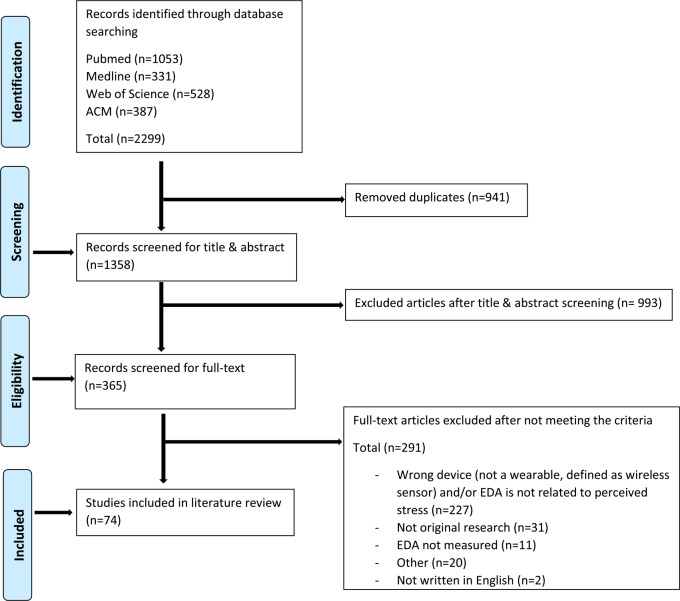
Flow chart diagram based upon Tricco *et al*. (2018).

In total, 2299 records were identified, of which 941 were duplicates (see Fig. 1 for an overview). After the screening of the title and abstract, 993 articles were excluded. The remaining 365 articles were assessed for eligibility by reading the full text, after which 291 were excluded. Reasons for exclusion in the full-text phase were as follows: no wearable device was used and/or EDA was not related to perceived stress (*n* = 227); EDA was not measured (*n* = 11); not original research (*n* = 31); not written in English (*n* = 2); and other reasons (*n* = 20; such as no full-text availability). Finally, 74 articles fulfilled the required criteria to be included in this scoping review. The full data extraction table of all 74 studies can be found in the appendix 1.

### Study population

Table 3 presents a summary of main study characteristics. The majority of studies (67.6%) measured stress exclusively on healthy subjects. 12.2% gave insufficient or unclear information about their study population. 9.5% reported stress measurements within children, aged from 0 to 17 years. Two of them included children diagnosed with ASD (Baker *et al.*, 2018; Goodwin *et al*., 2019) or other neurodevelopmental problems (Betancourt *et al*., 2017). Redd *et al*. (2020); Shao *et al*. (2020) and Weyn *et al*. (2022) included elementary school students in their research. One study included neonates on mechanical ventilation (Walas *et al*., 2020). 5.4% of the studies, realised by Melander *et al*. (2017), Lai Kwan *et al*. (2019), Deutsch *et al*. (2021) and Iaboni *et al*. (2022) studied patients diagnosed with dementia. 32.4% of the studies reported the inclusion of students, one of them including 62 participants with 8 having a mental illness (Schlier *et al*., 2019). 2.7% of the studies included patients with psychiatric problems (de Looff *et al*., 2019; Goodwin *et al*., 2019) and another 2.7% included subjects with a substance use disorder (Carreiro *et al*., 2020; Alinia *et al*., 2021). 8.1% of the studies included subjects under stressful circumstances, such as post-operative pain (Aqajari *et al*., 2021), work-related stress (Phitayakorn *et al*., 2015; Georgiou *et al*., 2019; Elsadek *et al*., 2020; Hosseini *et al*., 2022) and fear of spiders (Ihmig *et al*., 2020). Cella *et al*. (2019) included patients with psychotic episodes for their study, and Kuijpers *et al*. (2012) worked with one female schizophrenic patient in her early 20s.

**Table 3.** Summary of study characteristics of the included studies (*n* = 74)

Population characteristics	% of included studies (*n* = 74)	Wearable and stress characteristics	% of included studies (*n* = 74)
**Sample size**		**Context of stress evalulation**	
10 or less	20.3%	Challenging Behaviour	13.5%
11–30	43.2%	Healthcare	14.9%
31–100	24.3%	General stress detection (e.g. driving, job or task stress)	71.6%
More than 100	10.8%		
NA	1.4%	Locations of EDA wearable*	
**Gender (male) of study population**		Wrist	67.6%
Less than 40%	18.9%	Fingers	24.3%
40%–60%	29.8%	Palmar side of the hand	8.1%
More than 60%	24.3%	Other	5.4%
NA	27.0%	**Processing of EDA**	
**Study population**		Real time	5.4%
Healthy subjects	67.6%	Post-processing	94.6%
Children with ASD or neurodevelopmental disorder	4.1%	**Field or lab study**	
		Lab	56.7%
People with dementia	5.4%	Field	39.2%
Other (sub)clinical populations	10.7%	Combination	4.1%
Population not clearly defined	12.2%	**Stressor**	
**Range of mean age**		Induced	64.9%
0–17	9.5%	Observed	33.7%
18–40	60.8%	Combination	1.4%
41–59	5.4%	**Type of stress assessment**	
60+	5.4%	Self-report	79.7%
NA	18.9%	Observation of stress-related behaviour	12.2%
*NA = Not available, EDA = Electrodermal Activity,* *ML = Machine Learning.* *not multually exclusive (i.e. several bodily locations can be used within one study)			
		Combination	8.1%
		**Reported relation between stress and EDA**	
		Used EDA features to predict stress with ML	46.0%
		Direct correlation EDA – stress with other statistics than ML	27.0%
		Shown an indirect EDA – stress correlation	13.5%
		No correlation between EDA and stress found	8.1%
		No statistical analysis done	5.4%

Sample sizes of included studies varied between 1 and 1002. The majority of the studies (63.5%) included 30 or less participants. 10.8% of the studies included over 100 participants, 4.1% had one subject (Kuijpers *et al*., 2012; Vila *et al*., 2019; Hewgill *et al*., 2020) and 1.4% did not indicate a number of included subjects (Seoane *et al*., 2014). When looking at the gender distribution, 24.3% of all included studies included more than 60% males, 18.9% included less than 40% males and 29.7% were more or less gender balanced with 40–60% males. 27.0% of the studies did not report the gender of the participants.

Overall, the age of participants of the included studies varied between 0 and 93 years. 9.5% of all studies included children (0–17 years). Most studies (60.8%) included participants between 18 and 40 years of age on average, with most of them (50% overall) being mainly participants in their 20s. 5.4% of reported mean age ranges including participants over 60 years, and 8.1% of all studies in total included people above 60 in their age range.

### Wearables, bio-signals and their reported outcomes

#### Wearables

Sixteen different wearable devices were identified throughout the included studies, of which the most commonly used was the Empatica wristband in 40.1% of the articles, followed by the Shimmer3 sensor used in 6.8% of the studies (Xu *et al*., 2015; Williams *et al*., 2020; Wulvik *et al*., 2020; Giorgi *et al*., 2021) and Affectiva Q senor, which was used in 5.4% of the studies (Volonte *et al*., 2016; Betancourt *et al*., 2017; Baker *et al.*, 2018; Sano *et al*., 2018). Altogether, 67.6% of studies used a wristband sensor, 2.7% (Seoane *et al*., 2014; Lee *et al*., 2020b) used a glove with integrated sensors, 24.3% articles described devices with electrodes placed on the fingers, often with Velcro, for example, the Shimmer3 sensor, Biopac systems and the E243 inVivo metric systems corp. Marko (2016), Hollis *et al*. (2018) and Bruun *et al*. (2021) choose the Neurobit Optima 4 (finger electrodes), EmVibe (finger electrodes) and Mindplace Thoughstream (palmar electrodes), respectively, all of which were devices that needed to be carried (i.e. not attached to the body). 8.1% of the studies measured EDA on the palmar side of the hand, 2.7% measured on the chest (using the RespiBAN Professional; Jambhale *et al*., 2022) and torso (custom build garment; Hewgill *et al*., 2020). Finally, Betancourt *et al*. (2017) measured EDA on the ankle using the affective Q sensor.

About half of the field studies used the Empatica E4 wristband (56.3%). The wristbands DTI2 (Melander *et al*., 2017) and Empatica E4 (Deutsch *et al*., 2021; Iaboni *et al*., 2022) were used in clinical settings with patients with dementia. Additionally, Lai Kwan *et al*. (2019) attempted to detect ’significant moments’ in patients with dementia in a clinical field setting. De Looff *et al*. (2019) studied the aggressive behaviour of inpatient forensic psychiatric patients, and Goodwin *et al*. (2019) also analysed aggression in psychiatric ASD inpatients. Walas *et al*. (2020) and Aqajari *et al*. (2021) conducted an observational study to pain. In children, bodily measurement locations used were the wrist (Betancourt *et al*., 2017; Baker *et al.*, 2018; Goodwin *et al*., 2019; Redd *et al*., 2020; Weyn *et al*., 2022), fingers (Shao *et al*., 2020), palm (Walas *et al*., 2020) and ankle (Betancourt *et al*., 2017).

#### Bio-signals and processing of EDA

Besides the detection of EDA, the majority of studies (82.4%) simultaneously analysed other bio-signals such as HR, skin temperature (ST), respiration rate (RR), accelerometry, cortisol level, blood volume (BV), inter-beat interval (II) or other.

When analysing EDA, most included studies processed both the slow-changing component, the so-called skin conductance level (SCL), and the fast-changing component, the so-called skin conductance response (SCR). The SCL is related to a general arousal level, whereas SCR is a peak in EDA representing phasic activity, linked to for example novel or unexpected stimuli (Boucsein, 2012). However, no gold standard exists for the decomposition of the EDA signal. Included studies showed variation in EDA components used for analysis in terms of features extracted from SCR, SCL or EDA. Additionally, the method of decomposition of the EDA signal varies. Finally, algorithmic specifications or software licences used to process EDA and extract features were scarcely mentioned. Most studies (94.6%) processed the EDA signal offline, and 5.4% studies processed their data in real time (Vila *et al*., 2019; Anusha *et al*., 2020; Dissanayake *et al*., 2022; Hosseini *et al*., 2022).

#### Stressors studied

**Fig. 2.**
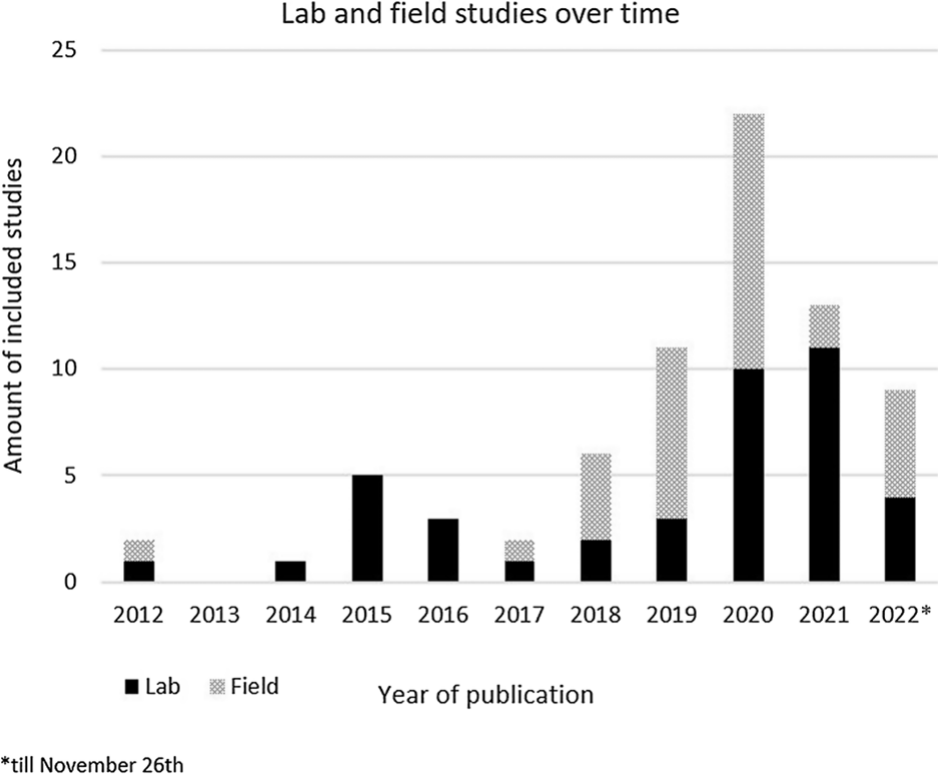
Lab and field studies over time relating electrodermal activity measured with a wearable and perceived stress.

The majority of studies (71.6%) were conducted in the context of general stress detection, such as job stress (e.g. Hosseini *et al*., 2022), drivers stress (e.g. Oh *et al*., 2021) or the development of a general stress detection algorithm (e.g. Jambhale *et al*., 2022). Furthermore, 14.9% of the studies were conducted in the context of healthcare, such as predicting pain intensity in post-operative patients (e.g. Aqajari *et al*., 2021). Finally, 13.5% of all studies were conducted in the context of challenging behaviour such as detecting ‘combat behaviour’ in people with dementia (Deutsch *et al*., 2021) or psychotic behaviour (Schlier *et al*., 2019). Studies were categorised on whether the stressor was induced (i.e. manipulated) or observed. 66.3% of the studies induced their stressor, for example, a surgical simulation (Georgiou *et al*., 2019) or watching film clips inducing emotions (Wang *et al*., 2020). 35.1% of the studies examined EDA in the context of observed (i.e. non-manipulated) stressors, such as agitated behaviours on a psychiatric ward of a nursing home (Iaboni *et al*., 2022). 56.7% of included studies were performed in a laboratory setting, 39.2% were field studies and 4.1% combined the laboratory setting with a field study (de Arriba Pérez *et al*., 2018; Kyriakou *et al*., 2019; Can *et al*., 2020). When considering the year of publication, a rapid increase of field studies is seen, especially over the last 5 years (see Fig. 2).

#### Relationship between EDA and perceived stress or stress-related behaviours

In 8.1% of the studies, no relationship between EDA and perceived stress was found. 5.4% of the studies described the relationship between EDA and perceived stress qualitatively. The remaining 86.5% of the included studies showed a (positive) association between EDA and perceived stress. Of those studies, 40.6% reported the accuracy (i.e. percentage of correct classifications regarding stress), which ranges between 42 and 100%, with an average of 82.6%. All studies conducted in the context of challenging behaviour reported a relationship between features of EDA and perceived stress, such as an increase in EDA features 20 min before the onset of aggression in people with dementia (de Looff *et al*., 2019) and EDA features combined with other bio-signals (such as heart activity, movement and ST) were successful in prediction agitation in people with dementia (Iaboni *et al*., 2022). In most studies using machine learning (ML), EDA features were combined with other features, making it not possible to draw conclusions about the independent ability of EDA features to predict perceived stress. 32.4% of studies using ML showed that stress can be predicted by using EDA features alone. Reported accuracies of classification models using other features next to EDA ranged from 74.2 to 100% with an average of 87.7%, whereas reported accuracies of classification models using solely EDA features ranged from 42 to 94% with an average of 71.2%. 13.5% of all studies only studied the relationship between EDA and perceived stress indirectly, by showing that features of EDA and perceived stress were increasing simultaneously, but not relating them directly.

**Fig. 3.**
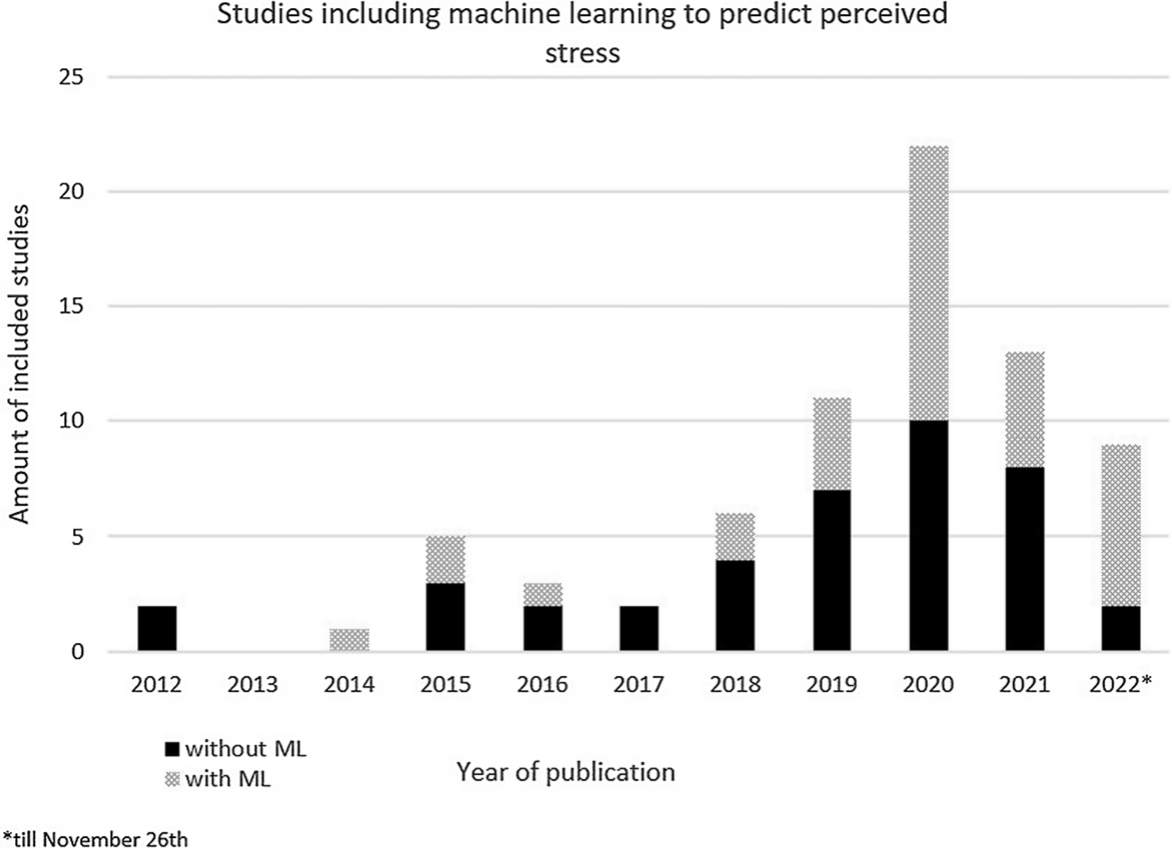
Machine Learning (ML) studies over time relating electrodermal activity measured with a wearable and perceived stress.

Stress assessment, in the form of self-report of perceived stress or the observation of stress-related behaviour (such as challenging behaviour), differed across studies. Additionally, the way of synchronising EDA features with perceived stress or stress-related behaviours differed between studies. 12.2% of the studies synchronised the perceived stress with the measured EDA signals continuously, others measured perceived stress before, during, after and/or in intervals during the performance of different tasks, experiments or the observation. In every study, either a self-reported stress questionnaire (e.g. State Trait Anxiety Inventory (STAI)), a behavioural analysis or a survey was performed. 46.0% of all included studies used ML to predict stress using EDA features next to other bio-signals, the majority (>50%) of them were conducted after 2020 (see Fig. 3). All of the algorithms used implemented EDA features in their classification model. 61.7% of all studies using ML were conducted in research populations aged 18–40 years, and 8.8% in 40–60 year old research populations, 8.8% in 60+ years of age research populations, 2.9% in children, and finally 17.6% of the studies using ML did not report the age of their research population.

Melander *et al*. (2017) showed a correlation between SCL and the prediction of agitation (73%), and also Iaboni *et al*. (2022) showed that agitation could be predicted with a high accuracy when ML was used, combining EDA features with other physiological variables (measured by the Empatica E4). To assess perceived stress, most studies used a measure of self-report (87.8%), of which the STAI was frequently used (21.5%), as were single items using Likert Scale (16.9%) and Self-Assessment Manikin and Profile of Mood States (6.2%). Of all studies using observations to assess stress, 42.8% studies analysed their findings via video analysis, looking at stress-related behaviour such as agitation (Lee & Chung, 2016; Betancourt *et al*., 2017; Kyriakou *et al*., 2019; Lai Kwan *et al*., 2019; Lee *et al*., 2020a; Iaboni *et al*., 2022).

Georgiou *et al*. (2019) and Hosseini *et al*. (2022) stated that the wristband could be accepted to provide accurate information about stress. An increase of EDA could be observed during increased anxiety (e.g. Georgiou *et al*., 2019; Tao *et al*., 2022), and ML algorithms are capable of predicting stress in real-life settings (e.g. Hosseini *et al*., 2022). Melander *et al*. (2017) suggested that the Philips DTI-2 wristband sensor can correctly predict 73.5% of agitation, before the caregivers’ behavioural observation. It was also found that data collected by the sensor were good for the visualisation of the perceived signals, supporting the nursing staffs’ observations and helping for a better understanding of the results. As for the studies conducted with children, Baker *et al*. (2018) stated that low EDA is related to externalising behaviour problems. Kuijpers *et al*. (2012) found an increased SCL before the onset of aggressive behaviour in their study. In contrast, Betancourt *et al*. (2017) faced many challenges to be addressed when measuring EDA in the field. Also in the lab, data were lost due to sensor failure. For example Bruun *et al*. (2021) had to discard data from 16 out of 61 participants due to sensor failure using the Mindplace ThoughtStream. Finally, it is shown that EDA features can be used not only to predict momentary stress but also daily or even monthly stress (Can *et al*., 2020).

## Discussion

Stress management plays an important role in health and care of people with stress-related physical (Deary *et al*., 2007) and/or behavioural problems (Matson *et al*., 2007; Melander *et al*., 2017). As insight into stress levels may facilitate stress management, the aim of this scoping review was to collect and summarise knowledge about currently existing wearable technologies measuring EDA designed to detect perceived stress. Seventy-four articles, published between 2012 and 2022, were identified in this study. The majority of the studies were performed in a laboratory setting with an induced, that is, manipulated, stressor, in a research population that was mostly young and healthy. In most studies, sample sizes were small (<30). The technologies used were predominantly wristbands, which, next to EDA, measured other physiological parameters. Most studies processed EDA offline (i.e. post-processing) and included slow changing (SCL) and rapid changing (SCR) features. The exact (i.e. mathematical) way of extracting EDA features varied across studies. Finally, the exact method of assessing perceived stress (i.e. questionnaires and behavioural observation schemes used) varied considerably across studies.

In included studies, the exact software used to process and extract EDA features was barely mentioned, and only a minority of studies were conducted outside of the lab, although field studies are increasing over the years. Using wearables in daily life is relatively new and influences the way of measuring and processing data. In natural environments, data processing should account for different types of measurement artefacts compared to clean and standardised laboratory settings. Therefore, lab data and outcomes cannot be translated directly to daily life situations. To ensure ecological validity, it is of utmost importance to validate the stress measurements of wearables in natural environments (Smets *et al*., 2019).

Moreover, as the ultimate goal is to integrate wearable technologies in a healthcare context, it is important to include a diversity of target populations in research. This means that people with neurocognitive (e.g. dementia) and developmental disorders (e.g. autism) as well as people with stress-related physical complaints (e.g. medically unexplained physical symptoms; MUPS) should be included in research populations. In this scoping review, only 20% of the studies included (sub)clinical populations and only 28% was conducted in the context of health or challenging behaviour. Around half of the studies included ML to predict stress. However, only 8% included older persons and a similar number of studies explored younger persons with disabilities. Critically, older persons (Mannheim *et al*., 2019) and people with disabilities (Shew, 2020) are too often excluded from research and design of digital technology and are significantly underrepresented in datasets used to train ML algorithms (Park *et al*., 2021). A main consequence is that such newly developed technologies may not fit their needs (Mannheim *et al*., 2022), or even that ML algorithms may be biased in their assessment of populations that are not young and healthy (Mehrabi *et al*., 2019; Joyce *et al*., 2021).

An adequate detection of EDA would lead to a better stress identification, offering a variety of possibilities for its implementation in the healthcare department. In the study of Dias & Cunha (2018), wearable health devices were reviewed on their current state and likely progression in the following years. They concluded that these technologies can support healthcare but are currently still limited in many ways: existing wearables are not available everywhere, are too expensive and are not yet suitable for ambulatory usage. Nevertheless, the integration of these technologies has the potential to lower healthcare costs. It could, amongst other, facilitate a non-pharmaceutical approach in stress management, support caregivers in monitoring patients, facilitate an optimised therapy provision and overall improve the patient’s quality of life (Cahill *et al*., 2007). Furthermore, caregivers are acknowledged to ‘play an important role in monitoring patient acceptance of the device’ (Mahoney & Mahoney, 2010). If wearables are to fulfil this potential, we need more research including the perspectives of patients and their caregivers.

A limitation found in this scoping review is that the assessment of perceived stress was performed in many different manners: the exact proceeding method, such as a questionnaire and/or the behavioural observation, the exact time point, when perceived stress was actually measured, and synchronisation with the continuous EDA measurements highly varied between studies. In addition, there is no gold standard for measuring stress, as various perceived stress scales were used. This hampers the comparability of the studies. As reported, stress was mostly assessed, before, after or in intervals during the performed study, an exact (i.e. real time) synchronisation and immediate comparison with the measured EDA signals was rare. Furthermore, not all studies directly compared perceived stress and EDA. Mostly, an offline analysis was done, while stress detection in daily life should be (pseudo)real-time to be relevant. The latter is influencing the possibilities of data processing, that is, offline data processing will be different from real-time data processing. This means that, in order to get an exact insight into the perceived stress of an individual in relation to their EDA levels during a so called ‘stressful’ task or situation, further investigation is needed considering real-time processing of measurements, also regarding the ML pipeline (Vos *et al*., 2022).

Interpreting skin conductance and thus detecting stress in real time on a personal level would have a high impact on healthcare. The development within the field of artificial intelligence (AI) can contribute greatly to this fact (see Vos *et al*. (2022) for a review). The use of ML has increased in the last years to detect stress using bio-signals, including EDA features, appreciating interpersonal variation. Studies like Anusha *et al*. (2020) and Iaboni *et al*. (2022) show that ML algorithms are very valuable when providing feedback regarding stress levels in a real-life setting, and stress could be detected before the caregivers observe it. Importantly, the ‘human in the loop’ remains crucial for annotating data (Melander *et al*., 2018; Iaboni *et al*., 2022) and interpretation of the measurement (Kikhia *et al*., 2016). During the development of AI applications, it is crucial that common biases such as sampling bias, aggregation bias or longitudinal data fallacy are mitigated (see Mehrabi *et al*. (2019) for an overview). Furthermore, the acceptance by healthcare professionals is of great importance for their use in practice. To ensure acceptance of AI-enabled solutions, ‘explainable’ AI (XAI) is paramount. Whereas traditional AI deals with the prediction or decision support by using ‘smart’ algorithms, XAI on top of that addresses the question ‘why is the AI predicting a certain outcome?’ XAI includes the application of unbiased, fair and transparent algorithms (Adadi & Berrada, 2018). Although future research is shifting towards this aim, this scoping review identified no articles focusing on XAI in the context of predicting perceived stress.

Another important factor in the acceptance and ultimate usage of wearables is the placement on the body. Studies included in this review used wearables mainly on the wrist, the hand or different fingers. Several studies have been performed to determine on which part of the body EDA can be detected in the best possible and most effective manner. A study from 2013 compared measuring EDA on the foot and fingers (Payne *et al*., 2013) and van Dooren *et al*. (2012) analysed 16 different bodily locations to measure EDA while provoking an emotional reaction. Both studies concluded that the fingers showed the best results when it comes to measuring EDA. However, finger sensors are used most often under laboratory conditions and not in daily life settings, limiting them in their application. Importantly, while jewellery-like rings have been introduced in the recent years (e.g. the Moodmetric smart ring (Vigofere Ltd, Finland), as described in Pakarinen *et al*., 2019), no studies using rings were found during the search of this scoping review, suggesting that despite their availability they are scarcely studied in relation to perceived stress. In addition, the level of evidence is only moderate for the fingers or the wrist to be the best placement option, suggesting focusing eventually more on other body parts such as the forehead, feet, shoulders, neck and chest. These bodily locations have shown good EDA responsiveness, with shoulders being the best solution (van Dooren *et al*., 2012). Subsequently, wearable technologies can be developed for these bodily locations, making them more suitable for ambulatory usage.

In addition to the placement on the body, also considering the exact context and especially the target group that is measured is important when selecting a sensor. For example, a wristband sensor in the form of a simple black watch might be a good solution when measuring in patients outside the lab. However, when working with people experiencing mental disorders such as dementia, there are some obstacles: the look of the watch-like wristband might confuse the patients and they would be able to either switch or take it off on their own. Furthermore, in case of a wrong placement of the device, signal processing might be disturbed and distorted. In the current review, the Empatica E4 wristband is the most often used tool in the included studies. Peeters *et al*. (2021) have shown that the use of this exact wearable is limited in real-life situations such as with people with dementia. This applies not only to the suitability of the wearable but also for the results regarding stress-related parameters, as well as the implementation of the wristband in daily life settings. Other technologies, such as ring sensors, could present similar issues in terms of causing confusion and interfering with daily life actions, making them not always suitable for use in practice. Less obtrusive tools, such as socks or patches, should therefore be further developed and analysed in future studies. However, the purpose of most of the studies included in this review was not to analyse the usability of the device, which made it difficult to assess their usability in real-life settings.

## Conclusion

Using wearables to measure features of EDA to predict perceived stress is promising as an assistive technique to support stress management. Nevertheless, research with people with health or care conditions and real-life settings is still limited. Additionally, studies conducting real-time stress prediction are scarce. Advances in ML are offering opportunities to account for interpersonal differences and apply real-time stress detection algorithms, using EDA features measured by wearables, under daily life conditions. However, to avoid biased applications, future research should include daily life situations, samples from diverse populations, as well as usability testing.

## Supplementary material

To view supplementary material for this article, please visit https://doi.org/10.1017/neu.2023.19
